# Influence of immunosuppression on the progression of experimental periodontitis and on healthy periodontal tissue: A rat in vivo study

**DOI:** 10.34172/joddd.2021.016

**Published:** 2021-05-05

**Authors:** Juliano Milanezi de Almeida, Henrique Rinaldi Matheus, Luiz Guilherme Fiorin, Elisa Mara Abreu Furquim, David Jonathan Rodrigues Gusman

**Affiliations:** ^1^Department of Diagnosis and Surgery, São Paulo State University (UNESP), School of Dentistry, Sao Paulo, Brazil

**Keywords:** Alveolar bone loss, Immunocompromised host, Periodontal diseases, Periodontitis, Rats

## Abstract

**Background.** The potent anti-inflammatory and immunosuppressive properties of glucocorticoids (GCs) might influence the progression of some disorders, such as periodontitis. Hence, this study aimed to investigate the influence of dexamethasone (DEX) on the alveolar bone loss (ABL) of healthy and periodontally compromised molars in rats.

**Methods.** Thirty male rats were randomly assigned to two groups: physiological saline solution (PSS) and DEX. The animals received subcutaneous injections of either 0.5 mL of PSS) (group PSS) or 2 mg/kg of DEX (group DEX) from one day before experimental periodontitis (EP) induction until euthanasia. EP was induced through ligature placement around the mandibular lower first molars at day 0. Contralateral molars remained unligated. Ten animals per period were euthanized on days 3, 7, and 14. Morphometric analysis was performed to access the ABL. Data were statistically analyzed with ANOVA followed by post hoc Tukey tests (*P* ≤ 0.05).

**Results.** Higher ABL was observed in both groups on days 7 and 14 than on day 3 (*P* ≤ 0.05). Concerning periodontitis, higher ABL was observed in group DEX on days 3, 7, and 14 days than group PSS at the same time intervals (*P* ≤ 0.05). Also, even in the contralateral unligated molars, group DEX exhibited higher ABL on days 3, 7, and 14 days than group PSS at the same time intervals (*P* ≤ 0.05).

**Conclusions.** Collectively, it can be concluded that DEX aggravates EP and induces spontaneous ABL in the healthy periodontium.

## Introduction

The concept of periodontitis as a simple bacterial infection is no longer accepted. It might be regarded as a combination of complex interactions between microbiota and a host.^1^ Even pathogenic microbiota resulting in subgingival dysbiosis, hosts’ immune, and inflammatory systems are critical determinants of the disease severity.^2^ The innate immune system is the first mechanism to suppress bacterial threats to the periodontium. However, when not effective, the breakdown reaches supporting periodontal tissues, including bone.^3^ The knowledge of the close relationship between inflammation and severity and extent of periodontitis provided new perspectives not only on possible targets for the management of the disease but also on the understanding of unassessed systemic conditions as risk factors for periodontitis.^2,4,5^

Glucocorticoids (GCs) are potent anti-inflammatory and immunosuppressive agents. Synthetic GCs have been widely used for many decades to treat a range of disorders, such as autoimmune, pulmonary, periodontal, and gastrointestinal diseases.^6^ These drugs are, in many cases, indispensable during the treatment of chronic diseases or following organ transplantation.^7^ However, the continuous use of steroidal anti-inflammatories has been listed as a risk factor for other illnesses, such as osteoporosis and periodontal diseases.^8,9^

Mainly on compromised periodontal tissues, animal experiments demonstrate that the use of corticoids can induce gingival ulceration, apical migration of the epithelium, attachment loss, and transseptal fiber disruption.^10,11^ On the other hand, in the healthy periodontium, no relationship has been reported in the literature so far.

As one of the components of the supporting periodontal tissues, bone biology is critical to the pathogenesis of periodontitis and maintenance of the periodontium. GCs directly impact bone, as they suppress the number, differentiation, and function of osteoblasts.^6^ This downregulation of the osteoblastic lineage might harm bone turnover and compromise the homeostasis in the periodontium. Mostafa et al^12^ suggested that gingival fibroblasts can be induced into osteogenic phenotype under an appropriate environment, such as osteogenic supplementation with an optimal dexamethasone (DEX) concentration. They also reported the dose-dependent effect of DEX, since the optimal osteogenic potential of the steroid on human gingival fibroblasts (HGF) was achieved at 0.1 and 0.5 µM concentrations. In contrast, a higher concentration of DEX downregulated the osteogenic effects.^12^

Even clinical trials to assess the influence of systemic GCs on the pathogenesis of periodontal diseases usually lack standardization.^13,14^ Therefore, further analysis and confirmation of this relationship are necessary in pre-clinical models.

Considering the necessity of knowledge in this field for proper periodontal maintenance and specific targeting on periodontal therapy, this study investigated the influence of DEX on the alveolar bone in the healthy periodontium and the severity of experimental periodontitis (EP) through a direct macroscopic analysis.

## Methods

### Animals

The experimental protocol was approved by the Ethics Committee on Animal Use under the code 01213-2012 in São Paulo State University (UNESP, School of Dentistry, Araçatuba). This research was undertaken following ARRIVE (Animal Research: Reporting of In Vivo Experiments).^15^ Sample size was calculated to achieve an 0.8 power and 0.05 alpha error based on a 12% potential standard deviation and the assumption that a 10% difference between groups/periods would be relevant, according to the National Centre for the Replacement, Refinement and Reduction of Animals in Research (NC3Rs).^16^ Forty-eight three-month-old Wistar rats (*Rattus norvegicus, albinus*) weighing 250–300 g were kept under 12-hour/12- hour light/dark cycles, 22±2ºC ambient temperature, 20 air changes per hour, and air humidity of about 55±5%. The animals were housed in plastic cages in groups of three and monitored daily, receiving feed and water ad libitum.

### Experimental model

This study followed a randomized, single-blind, controlled design. The animals’ tail was numbered from 1 to 48. The number sequence was uploaded to Minitab^®^ 17 software (Minitab Inc., State College, PA, USA). A blinded staff not involved in the study performed simple randomization (1:1 allocation ratio) using a computer-generated number table to the groups PSS 3 days, PSS 7 days, PSS 14 days, DEX 3 days, DEX 7 days, and DEX 14 days.

- Group PSS (n=15): One day before EP induction, the animals were given 0.5 mL of physiological saline solution (PSS) subcutaneously. The administration continued daily during the entire experiment. On day 0, EP was induced.
- Group DEX (n=15): One day before EP induction, the animals were given 2 mg/kg of DEX (Decadron, Aché Pharmaceutical Laboratories SA, Guarulhos, São Paulo, Brazil) subcutaneously.^17^ The administration continued daily during the entire experiment. On day 0, EP was induced.

### Anesthesia

The animals were anesthetized by a combination of ketamine hydrochloride (70 mg/kg) (Vetaset; Zoetis, Florham Park, NJ, USA) and xylazine hydrochloride (6 mg/kg) (Coopazine; Coopers, São Paulo, Brazil) intramuscularly to induce EP.

### Experimental periodontitis induction

On day 0, a #24 cotton thread was placed around the lower left first molar (Cotton Chain No. 24; Coats Corrente, São Paulo, SP, Brazil) to induce EP.^18,19^ The contralateral molars remained unligated to serve as controls.

### Euthanasia

Eight animals per group/period were euthanized with a lethal dose (150 mg/kg) of sodium thiopental (Cristália Ltda., Itapira, SP, Brazil) at 3-, 7-, and 14-day intervals after EP induction.

### Sample processing

The mandibles were collected and divided in the medial plane. Both the left (ligated) and right (unligated) hemi-mandibles were fixed in buffered 4% formaldehyde solution for 48 hours. The protocol for morphometry was adopted from Tatakis and Guglielmoni,^20^ as described by Corrêa et al.^21^ After gingival dissection, the specimens were immersed in 8% sodium hypochlorite for 4 hours and then washed in running water and dried. To distinguish the cementoenamel junction, the specimens were stained in 1% methylene blue. The stained specimens were appropriately oriented under a stereomicroscope, and digital images of the lingual and buccal aspects of the molars were obtained next to a millimeter-graduated ruler.

### Measurement of the alveolar bone loss

A single examiner, who was masked to the experimental groups and periods, carried out morphometric measurements. The measurements were performed using an image analysis software (Image Tool, University of Texas Health Science Center, San Antonio, TX). The software was used to calculate the exposed molar root surface area in mm^2^. Two measurements for each surface (lingual and buccal) were used to calculate the mean bone loss. The measurements of all the specimens were performed three times within a 7-day interval to assess the intra-examiner agreement between measurements.

### Statistical analysis

Data were analyzed using BioStat software (BioStat Version 5.0, Belém, PA, Brazil). Pearson’s correlation coefficient was used to calculate the agreement between measurements. Normality of alveolar bone loss (ABL) was analyzed using Shapiro–Wilk test followed by ANOVA and post hoc multiple comparisons with Tukey test (*P* ≤ 0.05).

## Results

The animals in group PSS exhibited no complications during the experiment. Their weight variation was within normal rates for healthy animals. The animals in group DEX showed gradual weight loss throughout the experiment, significantly higher than that in group PSS.

### Morphometric measurements of the ABL

**Figure 1:**
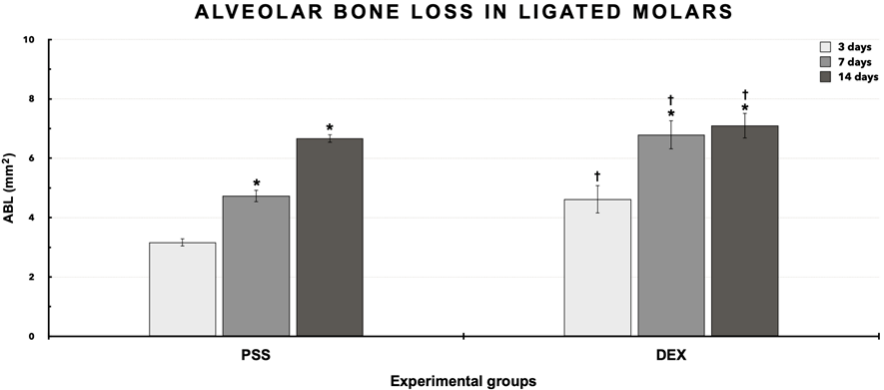
Graph showing the quantification (mean ± SD) of the alveolar bone loss (ABL) in ligated molars for each group and period. Statistical tests: ANOVA and Tukey. Symbols: *, statistically significant difference on day 3 in the same group (*P* ≤ 0.05); †, statistically significant difference with PSS at the same time intervals (*P* ≤ 0.05).

**Figure 2:**
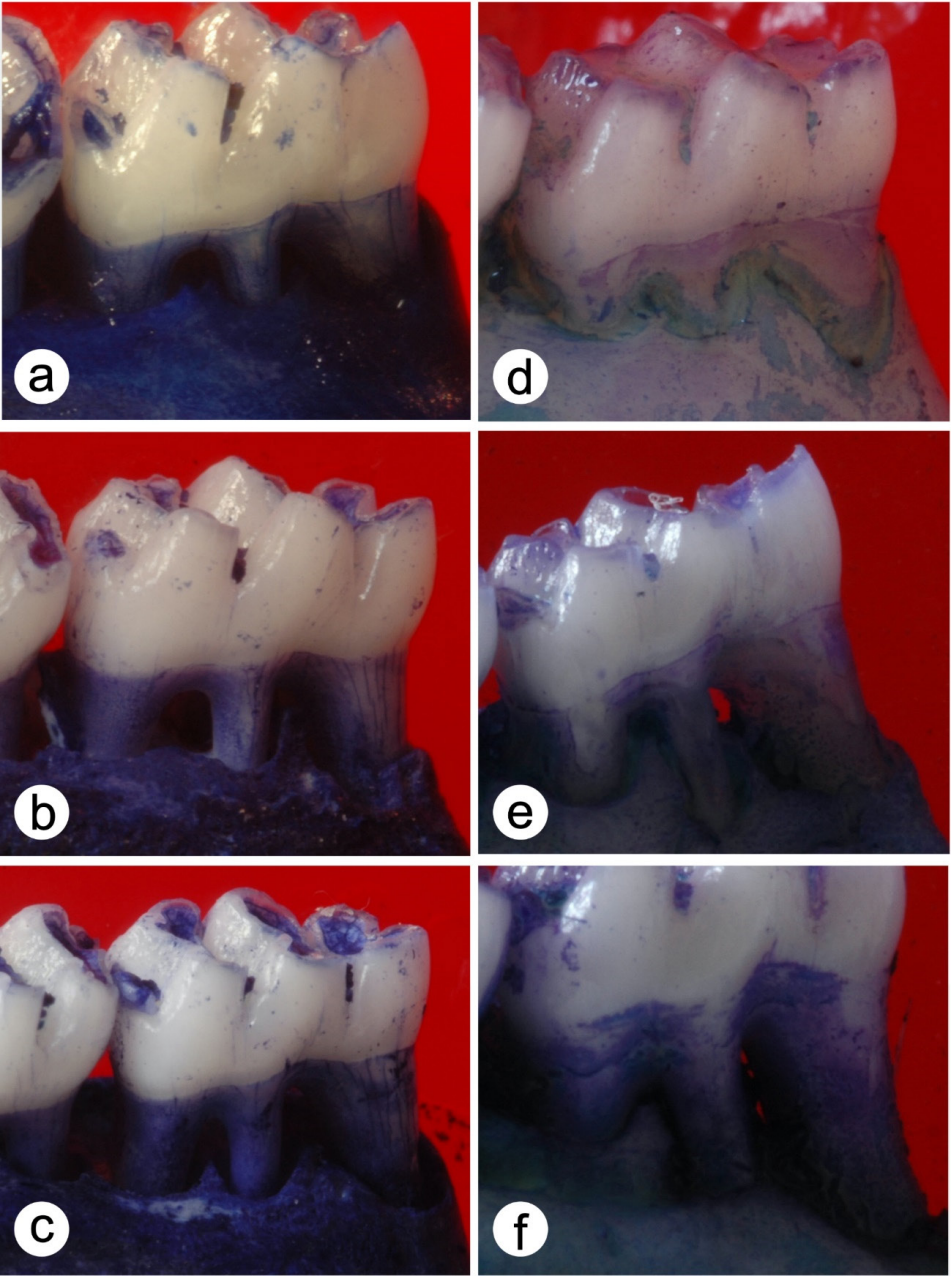
Representative photographs illustrating the morphometric findings of ABL of ligated molars in groups PSS-3d (a), PSS-7d (b) PSS-14d (c), DEX-3d (d), DEX-7d (e), and DEX-14d (f).

Pearson’s correlation coefficient revealed a 0.978 agreement rate between measurements (*P* ≤ 0.01). Figures 1 and 2 present the results (means ± standard deviations and images) of ABL for ligated molars. The intragroup analysis revealed significantly higher ABL (*P* ≤ 0.05) in groups PSS and DEX at 7 and 14 days compared to day 3 (*P* ≤ 0.05). Group DEX (Figure 2d, 2e, and 2f) showed higher ABL at 3 (4.62±0.470 mm^2^), 7 (6.79±0.48 mm^2^), and 14 days (7.10±0.43 mm^2^) compared with group PSS (Figure 2a, 2b, and 2c) at the same time intervals (3.17±0.13 mm^2^, 4.73±0.20 mm^2^, and 6.67±0.14 mm^2^, respectively) (*P* ≤ 0.05).

**Figure 3:**
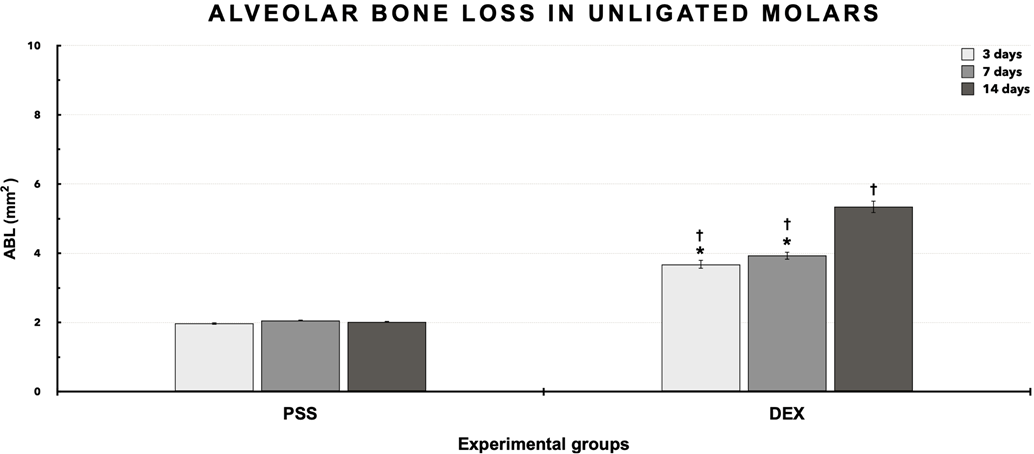
Graph showing the quantification (mean ± SD) of the alveolar bone loss (ABL) in unligated molars for each group and period. Statistical tests: ANOVA and Tukey. Symbols: *, statistically significant difference on day 14 in the same group (*P* ≤ 0.05); †, statistically significant difference with PSS at the same time intervals (*P* ≤ 0.05).

**Figure 4:**
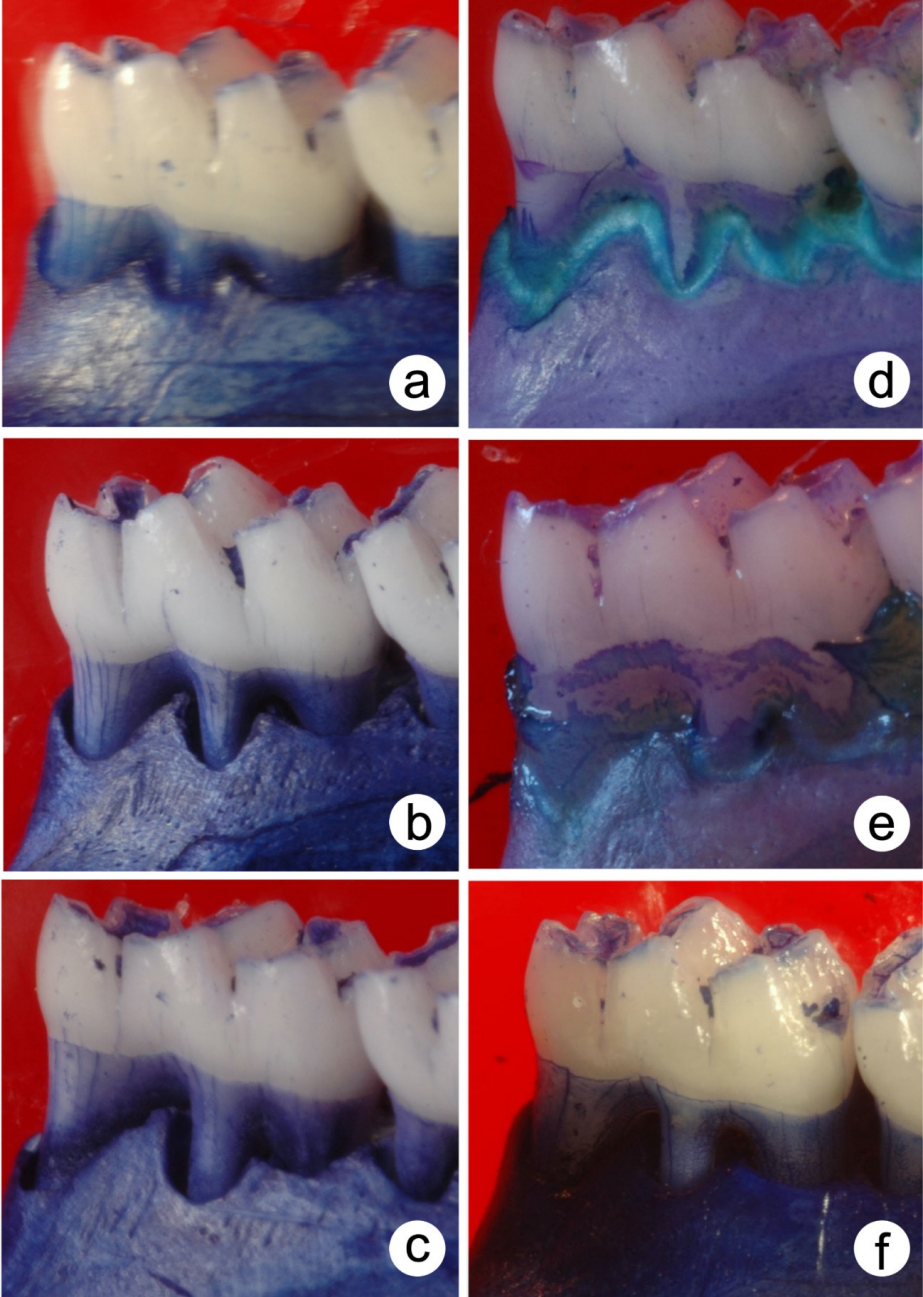
Representative photographs illustrating the morphometric findings of ABL of unligated molars in groups PSS-3d (a), PSS-7d (b) PSS-14d (c), DEX-3d (d), DEX-7d (e), and DEX-14d (f).

Figures 3 and 4 present the results (means ± standard deviations and images) of ABL for unligated molars. The intragroup analysis revealed significantly higher ABL (*P* ≤ 0.05) in the DEX group at 14 days than days 3 and 7 (*P* ≤ 0.05). Group DEX (Figure 4d, 4e, and 4f) showed higher ABL on days 3 (3.68±0.12 mm^2^), 7 (3.93±0.11 mm^2^), and 14 (5.34±0.18 mm^2^) than group PSS (Figure 4a-4c) at the same time intervals (1.97±0.03 mm^2^, 2.06±0.02 mm^2^, and 2.02±0.02 mm^2^, respectively) (*P* ≤ 0.05).

## Discussion

The degree of periodontal breakdown can be influenced by different factors affecting the pathogenesis of periodontitis. Systemic conditions might determine acceleration and aggravation in the course of periodontitis.^22^ Hosts’ immune and inflammatory systems play critical roles in the progression of periodontal diseases throughout the periodontium. GCs are frequently used in modern medicine due to their potent anti-inflammatory and immunosuppressive properties. Some evidence has already been highlighted concerning the relationship between GCs and periodontitis^14,15^; however, further evidence is necessary. The direct morphometric measurements presented by this research confirm that DEX not only aggravates the ABL in the presence of EP but also induces spontaneous ABL in non-periodontally compromised molars.

Animal models might bring the experimental design as close as possible to the clinical scenario. Ligature-induced periodontitis is a highly reproducible model that has been widely used to evaluate the progression of periodontitis.^23^ The pathogenesis of EP in this model is similar to humans since the cotton ligature leads to plaque accumulation, flattening and displacement of gingival crests, the proliferation of the epithelium into the underlining connective tissue, and initial penetration of mononuclear inflammatory cells.^24^ The initiation and persistence of plaque-induced periodontitis were confirmed by the presence of plaque and clinical signs of inflammation (e.g., edema and redness) in the gingival tissue of the animals at the time of euthanasia. These clinical findings were exacerbated in the DEX group.

Studies indicate that ABL can be accurately quantified through morphometric measurements, histometry, and micro-computed tomography, with no significant difference in the outcomes.^25,26^ Animal experiments in which ABL is the primary outcome parameter, morphometric measurements are easy to perform and provide cost-effective benefits over histometry and allow direct visualization of the defect, without interference from the cutting procedures for histological sections.^27^

The animals treated with DEX exhibited lethargy, hematoma, and alopecia at the time of euthanasia. Also, the drug-related decreased gastrointestinal absorption of nutrients led to weight loss over time in animals in the DEX group.^28^ These effects have been reported by other authors and might have affected our results because DEX administration simulates the long-term (3 to 4 years) treatment in humans.^29,30^

Concerning ligated molars, the DEX group exhibited significantly higher ABL than group PSS in all the experimental intervals. The negative impact of GCs on bone might be one of the factors leading to increased ABL in the DEX group since Bouvard et al^9^ reported their suppression during bone formation and increase during bone resorption. Also, GCs downregulate osteoprotegerin (OPG), an activator of the receptor activator of the nuclear factor kappa-Β ligand (RANKL) that potentially increases the activity of osteoclasts.^6^ Bostanci et al^31^ reported that RANKL and OPG were oppositely regulated in gingival crevicular ﬂuid in periodontitis patients. Notably, RANKL/OPG ratios were signiﬁcantly elevated in the gingival crevicular ﬂuid of three forms of periodontitis. RANKL/OPG ratio in tissues appears to be a significant indicator of potential bone resorption.^32^ The plausible harm in the ratio of RANKL and OPG caused by GCs, as well as their confirmed increased osteoclast activity and reduced bone turnover,^33^ are reliable reasons for the higher ABL in ligated and unligated animals of the DEX group.

One previous study reported *in vitro* osteogenic differentiation and mineralized matrix formation in human periodontal ligament cells treated with DEX.^34^ Osteogenesis can also be achieved by influencing growth factors, such as bone morphogenetic protein 2 and DEX on pluripotent cells.^35^ On this topic, Mostafa et al^12^ suggested that the treatment of HGFs with an optimal concentration of DEX is a potential source of cells for cell-based therapy for periodontal bone regeneration. On the other hand, a clinical trial failed to demonstrate this positive effect of GC on the clinical parameters of periodontitis in patients with neurological conditions.^36^

In addition, while considering the isolated influence of GCs on bone biomarkers, these drugs decrease the expression of osteocalcin and procollagen I N-terminal propeptide,^37,38^ both important regulators of osteoblast differentiation.^39^ Systemic GCs are also related to damages to hierarchical bone structure, such as glucocorticoid-induced osteoporosis.^33,40^ This influence is widely speculated and seems to target bone through many paths. Its negative impact on native bone was confirmed by the higher ABL in the healthy periodontium of the molars in the DEX groups compared to the PSS group at all the experimental intervals.

Animal experimentation aims to mimic the distinct conditions of the clinical scenario. Different protocols demand careful interpretation of the results provided by research. No clear relationship has been reported between DEX and ligature-induced periodontitis, which might be due to methodological variations.^11,29^

The interesting and elucidative data presented by this research should be interpreted with caution due to limitations inherent to any animal experiment, such as the different metabolic rates between animals and humans. This experiment might also encourage further investigations on specific biomarkers affected by immunosuppressive scenarios and expressed in periodontal tissues. Additionally, further experiments might assess therapeutic approaches or adjunctive therapies capable of mitigating or even suppressing the adverse effects of immunosuppression on the periodontium.

## Conclusion

The long-term and continuous administration of DEX for treating chronic conditions reproduced by the present research increased ABL in periodontitis animals, aggravating the EP and inducing spontaneous ABL in the healthy periodontium.

## Authors’ Contributions

All the authors have made substantial contributions to the concept and design of the study. JMA was involved in the conception, design, data analysis and interpretation, and drafted and critically revised the manuscript. All authors have given final approval of the version to be published.

## Acknowledgments

The authors thank the Department of Diagnosis and Surgery—Division of Periodontics, São Paulo State University (UNESP), School of Dentistry, Araçatuba, São Paulo, Brazil.

## Funding

No financial support is related to this study.

## Competing Interests

The authors have no conflict of interests.

## Ethics Approval

All applicable international, national, and institutional guidelines for the care and use of animals were followed. The experimental protocol was approved by the Ethics Committee on Animal Use under the code 01213-2012 of São Paulo State University (UNESP, School of Dentistry, Araçatuba), and conducted following the ARRIVE guidelines.
